# Treatment With the Shaker Head‐Lift Exercise in Head and Neck Cancer Patients With Radiation‐Induced Dysphagia: 12‐Month Results on Swallowing Function From a Randomized, Controlled Trial

**DOI:** 10.1002/hed.70352

**Published:** 2026-06-14

**Authors:** Signe Rödseth Smith, Caterina Finizia, Kerstin Petersson, Lisa Tuomi

**Affiliations:** ^1^ Department of Otorhinolaryngology, Head and Neck Surgery Sahlgrenska University Hospital, Region Västra Götaland Gothenburg Sweden; ^2^ Department of Health and Rehabilitation, Institute of Neuroscience and Physiology, Sahlgrenska Academy University of Gothenburg Gothenburg Sweden; ^3^ Department of Otorhinolaryngology, Head and Neck Surgery, Institute of Clinical Sciences, Sahlgrenska Academy University of Gothenburg Gothenburg Sweden

**Keywords:** deglutition disorders, head and neck neoplasms, intervention‐study, radiotherapy, randomized

## Abstract

**Background:**

Dysphagia is a common side‐effect after radiotherapy in head and neck cancer patients. This randomized study aimed to evaluate the effect of the shaker head‐lift exercise (HLE) after 12 months with flexible endoscopic evaluation of swallowing (FEES).

**Methods:**

Patients were randomly assigned to the intervention (*n* = 30) or the control (*n* = 31) group. The primary outcome was change according to the Penetration Aspiration Scale (PAS) at 12 months. Secondary outcomes included change according to the dynamic imaging grade of swallowing toxicity for flexible endoscopic evaluation of swallowing (DIGEST‐FEES) at 8 weeks and 12 months.

**Results:**

The intention‐to‐treat analysis revealed no significant difference between the intervention and control groups regarding the change in the PAS score from baseline to 12 months. Swallowing safety improved significantly in the control group at the 8‐week follow‐up.

**Conclusions:**

The HLE does not seem to improve swallowing function in individuals with radiation‐induced dysphagia.

## 1 Introduction

Head and neck cancers (HNC) comprise malignant tumors of the oral cavity, pharynx, larynx, paranasal sinuses, and salivary glands [1]. The global incidence of HNC is increasing by a predicted 30% annually [2]. In Western countries, this trend is attributed mainly to a rise in oropharynx cancers caused by human papillomavirus (HPV) infection [2]. Other primary risk factors for HNC are high tobacco and alcohol consumption [2]. Radiotherapy (RT) is a common treatment modality for HNC, recommended to some extent for around 80% of patients [3]. However, RT can lead to chronic adverse effects such as tissue fibrosis, atrophy, neuropathy, and sensory disorders, as it also impacts healthy tissues in the treated area [4, 5]. Dysphagia is a common side effect of RT, reported by 45% to as many as 85.5% of HNC survivors following treatment [6, 7, 8].

RT has several consequences for the structures involved in the different phases of swallowing. These include reduced tongue strength and tongue base retraction [9, 10], aberrant pharyngeal contractility due to thickening of the pharyngeal wall and constrictor muscles, reduced laryngeal elevation and reduced upper esophageal sphincter (UES) opening [9, 11, 12, 13], as well as stricture formation that reduces the opening of the UES [14]. Further, during maneuvers that engage the suprahyoid muscles, muscle strength is considerably lower in HNC patients compared to healthy individuals [15]. Altogether, these disorders can reduce swallowing function, undermining both swallowing safety and efficiency. A meta‐analysis showed that between 6 and 12 months after RT, invasion of bolus into the airway (i.e., penetration or aspiration) and bolus residue in the pharynx after swallowing occurred in around 30% and 70% of patients, respectively [12]. As such, impaired swallowing has a substantial impact on the health and life of HNC survivors, contributing to decreased food intake, weight loss, risk of malnutrition, reduced quality of life, and increased risk of aspiration pneumonia, a potentially fatal complication [7, 16]. The causes of dysphagia can vary over time after RT [17]. Acute toxicities present during RT can persist posttreatment with chronic inflammation and lymphedema being linked to posttreatment dysphagia, which can progress over time [4, 17]. Dysphagia can also debut later after a latency period, with this late sequelae being attributed to disturbed normal cellular behavior causing fibrosis, which in turn can lead to muscle and nerve dysfunction, including radiation‐induced neuropathies [4, 17]. Nevertheless, the exact mechanisms leading to the development or progression of dysphagia after RT are unclear [17].

Dysphagia rehabilitation seeks to improve long‐term swallowing function through a few different approaches, such as exercises to strengthen swallowing muscles, neuromuscular electrical stimulation (NMES), and skill‐based training focusing on timing, range of motion, and strength [18, 19, 20]. However, in the rehabilitation of RT‐induced dysphagia, randomized controlled trials (RCTs) of strengthening exercises have demonstrated only minor improvements or no positive outcomes [21, 22, 23], and an RCT evaluating NMES showed adverse results [20]. As such, there are currently no high‐quality empirical studies to inform the optimal type of swallowing exercise, timing of exercise, or exercise dose in the rehabilitation of dysphagia among HNC survivors [24, 25, 26]. There is a clear need for randomized studies with large sample sizes, high adherence rates, and validated tools to improve the quality of evidence [26, 27]. Another challenge is that a diverse range of outcome measures are used, which are limited in their capacity to capture certain aspects of swallowing function, making it hard to integrate results from different studies [20, 21, 22, 28]. Given that the causes and presentation of radiation‐induced dysphagia can evolve with time, it is also important to evaluate the effect of interventions through long‐term follow‐ups with instrumental assessment [12, 29].

The Shaker head‐lift exercise (HLE) was originally developed to strengthen swallowing muscles, such as the suprahyoid muscle involved in the anterior movement of the larynx, and was used subsequently to treat the reduced opening of the UES during swallowing in healthy older individuals [30]. Because the HLE addresses laryngeal elevation, which is commonly reduced after RT, it has become a widely used treatment method for dysphagia in HNC patients [31]. Nevertheless, evidence of its effectiveness is limited. A study in a small mixed cohort of HNC and stroke patients found a statistically significant reduction in post‐swallow aspiration in the HLE group compared with the traditional exercises group [32]. A later prospective clinical study in long‐term HNC survivors found that the HLE increased muscle strength; however, changes in swallowing function evaluated using the Penetration and Aspiration Scale (PAS) were inconsistent, and there was no improvement in hyoid‐bone movement or patient‐reported outcomes [15].

We initiated the current clinical study to address the lack of results from larger RCTs regarding the effect of the HLE on radiation‐induced dysphagia. To date, we have evaluated swallowing function by videofluoroscopy (VFSS), Flexible Endoscopic Evaluation of Swallowing (FEES), and the Eating Assessment Tool (EAT)‐10 after 8 weeks of intervention [33, 34] and health‐related quality of life (HRQL) after 12 months of maintenance training [35]. The results thus far show improvement in patient‐reported swallowing function, but no improvement in instrumentally assessed swallowing function at 8 weeks or HRQL at 12 months [33, 34, 35].

Since the publication of our initial results, the Dynamic Imaging Grade of Swallowing Toxicity for Flexible Endoscopic Evaluation of Swallowing (DIGEST‐FEES) has been validated for the Swedish HNC population [36]. This swallowing measure constitutes a single tool that describes the severity of dysphagia in the HNC population, facilitating the integration of results across studies. It is also the only available outcome tool that considers overall swallowing function observed during FEES and provides ordinal scales for swallowing safety (DIGEST safety), swallowing efficiency (DIGEST efficiency), and overall swallowing severity (DIGEST score) [28].

Here, we report the 12‐month swallowing function outcomes in our study of the HLE for radiation‐induced dysphagia in HNC patients. Specifically, we evaluated the change in swallowing function from baseline up to 12 months according to the PAS, EAT‐10, as well as the DIGEST‐FEES.

## 2 Materials and Methods

### 2.1 Participants

Adult patients with HNC of the oropharynx (tonsil, base of tongue), hypopharynx, and larynx who had undergone RT at Sahlgrenska University Hospital, Gothenburg, Sweden, 6 to 36 months prior were eligible for study inclusion. Patients were contacted by study SLPs and were offered a VFSS. To include participants with a range of swallowing difficulties from mild to severe, a PAS cut‐off score of at least 2 on two swallows during the VFSS was applied (Figure 1). After VFSS, 87 patients were invited to participate. Exclusion criteria were previous treatment for HNC, tracheostomy, laryngectomy, neurological or neuromuscular disease, surgery for HNC (except tonsillectomy), previous swallowing difficulties unrelated to HNC, inability to swallow any bolus, or inability to perform the HLE. Sixty‐one patients were included and randomized into either the intervention group (*n* = 30) or the control group (*n* = 31). The inclusion took place between 2010 and 2018.

**FIGURE 1:**
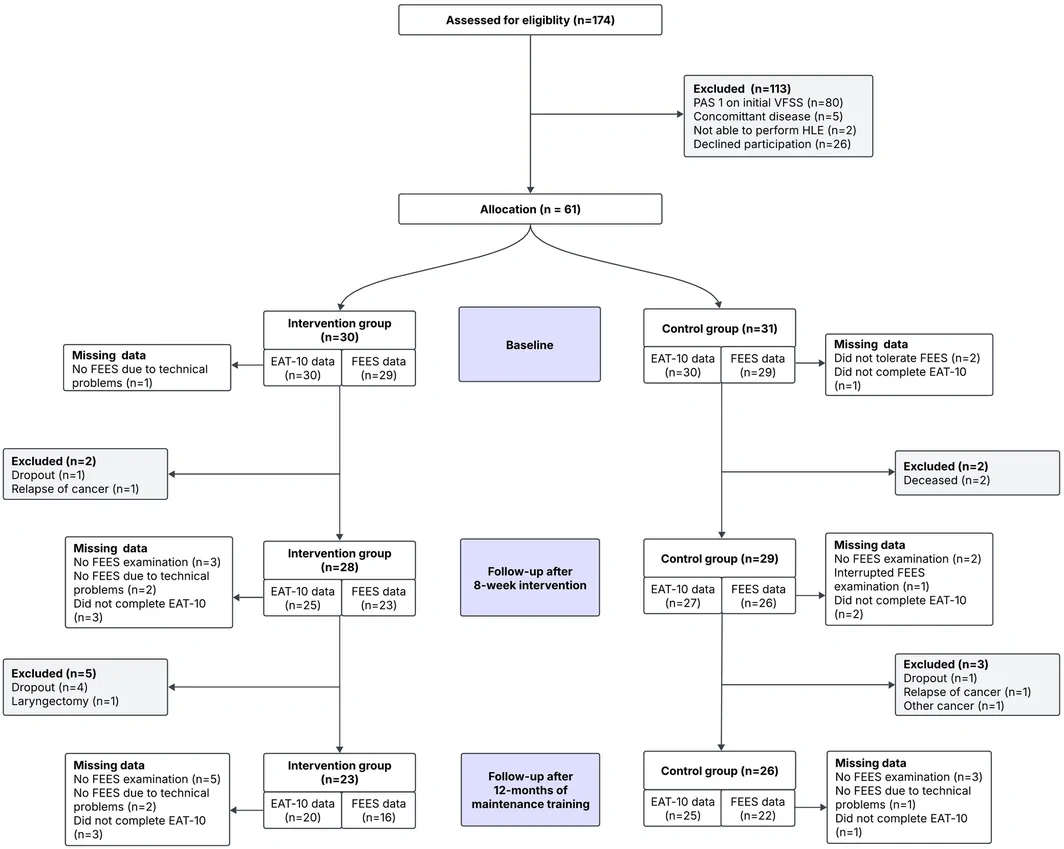
Flowchart of study participation and analyses. [Color figure can be viewed at wileyonlinelibrary.com]

### 2.2 Oncological Treatment

All participants received External Beam Radiation Therapy (EBRT) according to the regional program at the time of treatment. The EBRT delivery system was typically either intensity‐modulated or volumetric modulated arc therapy (IMRT or VMAT, respectively). The given dose for the primary tumor was typically 68 Gray (Gy) with 2 Gy delivered once daily, 5 days a week. Chemotherapy with cisplatin was given concomitantly (*n* = 39) or as induction (*n* = 9) to RT. Thirteen participants received no chemotherapy, and 20 participants received brachytherapy.

### 2.3 Study Design

Randomization was balanced according to tumor location, tumor stage, age, gender, comorbidities (Adult Comorbidity Evaluation‐27, ACE‐27), and the maximum PAS score on the initial VFSS. Swallowing function was evaluated by FEES and patient‐reported outcome measures at the 8‐week and 12‐month follow‐ups. The PAS and EAT‐10 results at the 8‐week follow‐up have been previously published [33] and are presented here to give further context to the changes in scores from baseline to the 12‐month follow‐up. Treatment and sociodemographic data, as well as eating‐related and nutritional data, were obtained through medical records or study‐specific questionnaires (Tables 1 and 2). A power calculation was performed on the PAS score to determine the sample size, where a 1‐point change in the PAS score was considered clinically significant. It was determined that 25 participants in each group were sufficient to afford a predicted power of 80% (Mann–Whitney *U*‐test, alpha = 0.05).

**TABLE 1:** Treatment and sociodemographic data for participants at baseline.

Variable	Intervention group (*n* = 30)	Control group (*n* = 31)	*p* ^b^
	Mean ± SD median (min‐max)	Mean ± SD median (min‐max)	
Age (years)	63.1 ± 9.1 63 (44–80)	63.8 ± 7.1 63 (49–75)	0.80
Time since end of radiotherapy (months)	10.7 ± 5.5 9.0 (6.0–29.0)	12.8 ± 7.9 9.0 (6.0–37.0)	0.52
	*n* (%)^a^	*n* (%)^a^	
Sex			0.57
Male	21 (70%)	24 (77%)	
Female	9 (30%)	7 (23%)	
Comorbidity (ACE‐27)			0.038
None	17 (57%)	11 (36%)	
Mild	11 (37%)	13 (42%)	
Moderate	2 (7%)	6 (19%)	
Severe	0 (0%)	1 (3%)	
Tumor location			0.92
Tonsil	13 (43%)	11 (36%)	
Base of tongue	11 (37%)	12 (39%)	
Larynx	4 (13%)	5 (16%)	
Hypopharynx	2 (7%)	3 (10%)	
Tumor stage			0.66
I	1 (3%)	4 (13%)	
II	5 (17%)	2 (7%)	
III	2 (7%)	3 (10%)	
IV	22 (73%)	22 (71%)	
Chemotherapy			0.91
None	6 (20%)	7 (23%)	
Concomitant	20 (67%)	19 (61%)	
Induction	4 (13%)	5 (16%)	
Radiotherapy			0.67
Conventional	28 (93%)	28 (90%)	
Accelerated	2 (7%)	3 (10%)	
Brachytherapy	11 (37%)	9 (29%)	0.53
Lives alone	8 (27%)	11 (36%)	0.58
Working	15 (50%)	17 (55%)	0.80
Education			0.40
Primary school	10 (35%)	10 (32%)	
High school	12 (41%)	10 (32%)	
College/university < 4 years	4 (14%)	5 (16%)	
College/university ≥ 4 years	3 (10%)	6 (19%)	
Smoking status			0.72
Never smoked	10 (33%)	7 (23%)	
Quit smoking ≥ 12 months ago	12 (40%)	16 (52%)	
Quit smoking < 12 months ago	4 (13%)	3 (10%)	
Current smoker	4 (13%)	5 (16%)	
Risk of use alcohol	3 (10%)	4 (13%)	1.00
Yes			
Xerostomia			0.71
No trouble with dry mouth	3 (10%)	2 (4%)	
Experience dry mouth but am not affected by it	2 (7%)	3 (10%)	
Experience dry mouth now and then	10 (33%)	14 (45%)	
Experience dry mouth always	15 (50%)	12 (39%)	

Abbreviations: ACE‐27 = adult comorbidity evaluation‐27; SD = standard deviation.

^a^ Percentage values are rounded; therefore, do not always sum to 100.

^b^ For tests between two groups, Fisher's exact test was used for dichotomous variables, the Mantel–Haenszel chi‐squared trend test for ordered categorical variables, the chi‐squared test for non‐ordered categorical variables, the Mann–Whitney *U*‐test for non‐normally distributed continuous variables, and the two‐sample *t*‐test for normally distributed continuous variables.

**TABLE 2:** Eating‐related and nutritional variables at baseline and the 12‐month follow‐up.

Variable	Intervention group (*n* = 30)		Control group (*n* = 31)			
	Baseline	12 months	Baseline	12 months	Baseline	12 months
	Mean ± SD median (min‐max)	Mean ± SD median (min‐max)	Mean ± SD median (min‐max)	Mean ± SD median (min‐max)	*p* ^b^	*p* ^b^
MIO (mm)	42.5 ± 8.4 41.0 (27.0–59.0)	36.7 ± 12.6 41.5 (4.5–51.0)	40.6 ± 10.8 42.0 (6.8–57.0)	38.5 ± 12.9 41.5 (4.6–55.0)	0.44	0.64
	*n* (%)^a^	*n* (%)^a^	*n* (%)^a^	*n* (%)^a^		
BMI class (1–4)					0.30	0.49
Underweight < 18.5	4 (13%)	1 (5%)	4 (13%)	4 (16%)		
Normal weight 18.5–24.9	22 (73%)	12 (60%)	18 (60%)	13 (52%)		
Overweight 25–30	4 (13%)	6 (30%)	7 (23%)	7 (28%)		
Obese ≥ 30	0 (0%)	1 (5%)	1 (3%)	1 (4%)		
Nasogastric tube/PEG	3 (10%)	2 (11%)	4 (13%)	1 (4%)	1.00	0.58
Pneumonia after disease	4 (13%)	1 (5%)	1 (3%)	0 (0%)	0.20	0.44

Abbreviations: BMI = body mass index; MIO = maximal interincisal opening; PEG = percutaneous endoscopic gastrostomy; SD = standard deviation.

^a^ Percentages rounded; therefore, does not always sum to 100.

^b^ For tests between two groups, the Fisher's exact test was used for dichotomous variables, the Mantel–Haenszel chi‐squared trend test for ordered categorical variables, and the two‐sample *t*‐test for continuous variables.

### 2.4 The HLE Intervention

The HLE intervention is performed in the supine position by raising the head high enough to see one's toes without raising the shoulders. It consists of both isometric and isokinetic head lifts and was originally developed to strengthen the suprahyoid muscles involved in the anterior movement of the larynx, subsequently to improve UES during swallowing [30, 37]. Participants in the intervention group received instructions from SLPs in performing the HLE, as well as video and written instructions. Over 8 weeks, these participants performed the HLE intervention three times a day; one treatment session consisted of three 60‐s static head lifts (isometric training) with a 60‐s pause in between each, followed by 30 consecutive head lifts (isokinetic training). Participants recorded their training in a training diary. For the first 2 weeks of training, participants met with a study SLP two times per week and were thereafter contacted every other week via telephone to monitor training. Over the following 12 months, participants were instructed to do at least three HLE sessions per week, preferably one session per day. Participants managed their maintenance training independently and recorded it in a training diary.

Participants in both the intervention group and the control group received conventional dysphagia management, which consisted of advice about food consistencies, eating tempo, bite size while eating, and head positioning, as well as maneuvers such as effortful swallow, Mendelsohn, and supraglottic swallow, to be used in a compensatory fashion. Further description of the control group management is found in previous publications from this RCT [33, 34].

### 2.5 Assessment and Endpoints

#### 2.5.1 Instrumental Evaluation of Swallowing

FEES was performed by experienced SLPs at baseline, after 8 weeks of intervention, and after 12 months of maintenance training. The equipment used differed throughout the study: an Olympus ENF‐P4 flexible endoscope (Olympus Inc., Japan) attached to a Wolf‐Type 5052 light source (Richard Wolf GmbH, Germany), an Olympus ENF‐VH videofiberendoscope attached to an Olympus Elite II OTV‐S200 light source (Olympus Inc.), or a Xion EV‐NC videofiberendoscope attached to a Xion Endoportable CFT‐003 dock (Xion GmbH, Germany). Digital video recordings were made using a video database system (IMIS, Atea AB, Sweden), Xion Endoportable CFT‐003, or by digitalization of analog VHS recordings.

Participants were offered boluses of different consistencies according to the International Dysphagia Diet Standardization Initiative (IDDSI) using the following protocol: 3, 5, and 10 mL of mildly thick liquid (IDDSI 2); 3, 10, and 20 mL of thin liquid (IDDSI 0); and one‐fourth of a biscuit dipped in mildly thick liquid (IDDSI 7) [38]. All boluses of liquid consistency were colored with green food dye and administered with a spoon or a cup. Boluses were swallowed on command, except the biscuit and the 20 mL bolus of thin liquid, which participants were instructed to drink at their own pace. Examiners were allowed to exclude boluses if there was a risk of harmful aspiration. Excluded boluses could be tested at follow‐up if deemed safe.

The main analysis of the FEES films was blinded and performed by one SLP. To measure intra‐rater reliability, 25% of the recordings were analyzed in duplicate by the same SLP. To measure inter‐rater reliability, a second SLP independently evaluated 25% of the recordings. The PAS was used as the primary endpoint measure in this study. The scale assesses two dimensions of swallowing function—depth of bolus into the airway and the swallower's response to the bolus entering the airway—according to one ordinal 8‐point scale. A PAS score of 1 indicates no bolus entering the airway, and a score of 8 indicates the bolus passing the vocal folds with no effort to expel it [39]. Supplementary descriptive results were produced using Steele's reorganization of the PAS into 4 categories that reflect increasing impairment: A (PAS score of 1, 2, and 4); B (PAS score of 3, 5, and 6); C (PAS score of 7); and D (PAS score of 8) [40].

As a secondary outcome measure, swallowing function was evaluated according to the DIGEST‐FEES protocol. The instrument was developed specifically for the HNC population and provides a valid and reliable measure of overall pharyngeal swallowing severity for this population [28]. It is based on three 5‐point ordinal scales, where 0 denotes no pharyngeal dysphagia, and 4 denotes life‐threatening pharyngeal dysphagia. A safety profile based on the PAS (DIGEST safety) and an efficiency profile based on the percentage of pharyngeal residue (DIGEST efficiency) are summarized to produce a global score (DIGEST score) [41].

#### 2.5.2 Patient‐Reported Outcomes

As a subjective evaluation of swallowing function, participants answered the Swedish version of the EAT‐10, which was validated for the Swedish HNC population in 2025 [42] and showed sufficient psychometric properties in HNC patients [42], as well as excellent validity, reproducibility, and internal consistency [43]. For the HNC population, the EAT‐10 has been shown to correspond well with functional oral intake and to indicate the presence of pharyngeal residue [44, 45]. The instrument comprises 10 items scored on a 5‐point Likert scale, where 0 suggests no problems, and 40 suggests severe problems [43].

### 2.6 Statistical Analysis

An intention‐to‐treat (ITT) approach, comprising all randomized participants (Figure 1), was used to analyze the primary outcome, the change in the PAS score from baseline to 12 months, and the secondary outcomes, which included the change in the PAS score from 8 weeks to 12 months, the EAT‐10 score from baseline to 12 months and 8 weeks to 12 months, and the DIGEST‐FEES scores from baseline to 8 weeks, 8 weeks to 12 months, and baseline to 12 months. For robustness, primary and secondary outcomes were also analyzed using complete case analysis and the per protocol population. The per protocol population was defined as all randomized participants with ≥ 75% adherence to the HLE training during the 8‐week intervention period, who had data from baseline and the 12‐month follow‐up (*n* = 40). Exploratory analyses were performed for two subgroups consisting of participants who received scores reflecting moderate‐to‐severe difficulties on instrumental assessment at baseline: PAS score ≥ 5 and DIGEST score ≥ 2. All exploratory analyses were performed on complete cases.

For the ITT analysis, multiple imputation was performed on 50 studies. The imputation model was constructed to include baseline patient characteristics and baseline values that were related to the change in and missingness of the outcome variable of interest. Each treatment group was imputed separately. All variables were modeled in one and the same model using PROC MI procedure in SAS using fully conditional specification with regression method. The seed 8753 will be used. The outcome variables for the change of the primary variables were imputed. The relationship between the primary and secondary outcome variables and continuous and ordered categorical variables was tested using Spearman correlation. For dichotomous variables, the relationship was tested using the Mann–Whitney *U*‐test. For non‐ordered categorical variables, the Kruskal–Wallis test was used.

In the comparison of groups regarding the primary and secondary outcomes, general linear models adjusted for baseline characteristics and baseline values were used for normally distributed variables. Ordered categorical variables were assessed using ordinal logistic regression adjusted for baseline values. Fisher's exact test was used for dichotomous variables. The Mantel–Haenszel chi‐square trend test was used for ordered categorical variables. The chi‐squared test was used for non‐ordered categorical variables. The *t*‐test was used for normally distributed continuous variables, and the Mann–Whitney *U*‐test was used for non‐normally distributed continuous variables.

Intra‐ and interrater reliability was estimated according to weighted kappa statistics, and results were interpreted as follows: ≤ 0, less than chance agreement; 0.0–0.20, slight agreement; 0.21–0.40, fair agreement; 0.41–0.60, moderate agreement; 0.61–0.80, substantial agreement; and 0.81–1.00, almost perfect agreement.

A significance level of 0.05 was applied. The primary analysis was the only confirmatory analysis. All statistical analyses were performed using SAS software version 9.4 (SAS Institute Inc., Cary, NC, USA).

### 2.7 Ethical Considerations

The study was approved by the Regional Ethical Review Board in Gothenburg, Sweden, and was conducted according to the Declaration of Helsinki. All participants gave their written informed consent before inclusion. The study population has been described in part before using instrumental assessment directly after intervention, and HRQL results have been reported at 12 months [33, 34, 35].

## 3 Results

Sixty‐one patients participated in the study. The participants in the intervention group and the control group were comparable regarding sociodemographic factors, disease characteristics, and oncological treatment, with no statistically significant differences at baseline (Table 1). However, there was a statistically significant difference between the groups at baseline on the ACE‐27, reflecting a higher level of comorbidity in the control group. The mean age of the participants was 63 years in both groups, and the most common tumor locations were the tonsils and the base of the tongue. There were no statistically significant differences between the intervention group and the control group regarding eating‐related and nutritional variables either at baseline or at 12 months (Table 2).

As previously reported, adherence to the HLE training during the 8‐week intervention period was high, with a mean rate of 89% [33]. However, adherence to training during the 12‐month maintenance period was low. Participants were instructed to perform one HLE session per day or at least three sessions per week. The mean percentage of weeks during which a minimum of three sessions were completed was 40.4%.

### 3.1 Penetration and Aspiration Scale (PAS)

There were no statistically significant differences in the change in the maximum PAS score when comparing the intervention and control groups from baseline to 12 months or 8 weeks to 12 months (*p* = 0.94 and *p* = 0.79, respectively; Table 3). Looking at descriptive data of within‐group changes, the mean PAS score deteriorated during the 8‐week intervention period for the intervention group and then improved during the 12‐month maintenance period, with an overall mean improvement between baseline and 12 months of 1 PAS level. Conversely, in the control group, the mean PAS score improved during the intervention period and deteriorated during the maintenance period, with an overall mean improvement between baseline and 12 months of 1 PAS level. Results from the sensitivity analysis using complete case analysis and the per protocol population did not differ from those using the ITT population. Supplementary descriptive analyses using Steele's categorization of PAS scores reflect a greater improvement in the control group as compared to the intervention group over all study periods (Table S1).

**TABLE 3:** Results of Penetration Aspiration Scale (PAS) scores and Eating Assessment Tool (EAT)‐10 scores for the control and intervention groups at baseline, 8‐week follow‐up, and 12‐month follow‐up, as well as comparison of changes between the control and intervention groups from baseline to 8 weeks, 8 weeks to 12 months, and baseline to 12 months.

Variable	Intervention (*n* = 30)						Control (*n* = 31)					
	Baseline	8 weeks	12 months	Δ Baseline—8 weeks	Δ 8 weeks—12 months	Δ Baseline—12 months	Baseline	8 weeks	12 months	Δ Baseline—8 weeks	Δ 8 weeks—12 months	Δ Baseline—12 months
	Mean ± SD median (min‐max)	Mean ± SD median (min‐max)	Mean ± SD median (min‐max)	Mean ± SD median (min‐max)	Mean ± SD median (min‐max)	Mean ± SD median (min‐max)	Mean ± SD median (min‐max)	Mean ± SD median (min‐max)	Mean ± SD median (min‐max)	Mean ± SD median (min‐max)	Mean ± SD median (min‐max)	Mean ± SD median (min‐max)
PAS	4.0 ± 2.4 3.0 (1.0–8.0)	4.2 ± 2.0 5.0 (1.0–8.0)	3.5 ± 1.9 4.0 (1.0–7.0)	0.2 ± 2.2 0.0 (−4.0–4.0)	−0.6 ± 2.3 0.0 (−4.0–4.0)	−1.0 ± 3.1 0.0 (−7.0–4.0)	4.8 ± 2.1 5.0 (1.0–8.0)	4.1 ± 2.4 3.5 (1.0–8.0)	3.7 ± 2.3 4.5 (1.0–8.0)	−0.8 ± 2.7 0.0 (−6.0–4.0)	0.0 ± 2.9 0.0 (−7.0–6.0)	−1.0 ± 2.3 −0.5 (−5.0–4.0)
EAT‐10	14.5 ± 10.1 13.5 (0.0–35.0)	10.6 ± 8.9 8.0 (0.0–30.0)	10.9 ± 9.3 9.0 (0.0–31.0)	−3.6 ± 6.6 −3.0 (−22.0–8.0)	0.7 ± 4.3 0.0 (−5.0–13.0)	−1.9 ± 5.7 −1.0 (−13.0–10.0)	14.3 ± 9.7 13.5 (0.0–33.0)	12.3 ± 9.7 11.0 (0.0–31.0)	9.0 ± 8.2 7.0 (0.0–25.0)	−1.5 ± 4.6 −1.0 (−8.0–13.0)	−2.0 ± 6.7 0.0 (−16.0–13.0)	−4.1 ± 7.1 −3.0 (−20.0–9.0)

Variable	Difference in change (Δ) between groups^a^		
	Baseline—8 weeks	8 weeks—12 months	Baseline—12 months
	Mean (95% CI) *p* value	Mean (95% CI) *p* value	Mean (95% CI) *p* value
PAS	0.37 (−0.86–1.60) *p* = 0.55	−0.18 (−1.53–1.17) *p* = 0.79	−0.06 (−1.62–1.50) *p* = 0.94
EAT‐10	−1.67 (−4.56–1.23) *p* = 0.26	1.85 (−1.28–4.97) *p* = 0.25	1.45 (−1.83–4.72) *p* = 0.39

*Note:* The PAS scale scores range from 1 (no bolus enters the airway) to 8 (bolus passes the vocal folds with no effort to expel it). The EAT‐10 total score ranges from 0 to 40 points with a higher value indicating worse function.

Abbreviations: CI = confidence interval; SD = standard deviation.

^a^ Analyses of primary and secondary outcomes were performed using general linear models adjusted for baseline characteristics and baseline values. Missing data were handled by multiple imputation including baseline variables related to the outcome and its missingness.

### 3.2 Eating Assessment Tool (EAT)‐10

No statistically significant differences were observed in EAT‐10 scores when comparing the intervention and control groups regarding changes from baseline to 12 months or 8 weeks to 12 months (*p* = 0.39 and *p* = 0.25, respectively; Table 3). Looking at descriptive data of within‐group changes, the mean EAT‐10 score improved by 3.6 points from baseline to 8 weeks and then deteriorated by 0.7 points from 8 weeks to 12 months in the intervention group. In the control group, the mean EAT‐10 score improved over all study periods. From baseline to 12 months, this resulted in an overall improvement in the mean EAT‐10 score by 1.9 points in the intervention group and 4.1 points in the control group (Table 3). Results from the sensitivity analyses did not differ from those of the ITT analysis.

### 3.3 The Dynamic Imaging Grade of Swallowing Toxicity for Flexible Endoscopic Evaluation of Swallowing (DIGEST‐FEES)

There was a statistically significant difference in the change in the DIGEST safety score when comparing the intervention and control groups from baseline to 8 weeks. In the control group, 56% of participants experienced an improvement, and 8% deterioration, as compared to only 18% showing an improvement in the intervention group and 27% deterioration (*p* = 0.033; Table 4). There was no statistically significant difference in the change in the DIGEST safety score between the groups from baseline to 12 months or from 8 weeks to 12 months. No statistically significant differences were observed in the change in DIGEST efficiency or overall DIGEST scores. Results from the sensitivity analyses did not differ from those of the ITT analysis.

**TABLE 4:** Comparison of dynamic imaging grade of swallowing toxicity for flexible endoscopic evaluation of swallowing (DIGEST‐FEES) safety and efficiency scores and overall DIGEST scores between the control and intervention groups from baseline to 8 weeks, 8 weeks to 12 months, and baseline to 12 months.

Variable	Intervention group (*n*) (%)^a^			Control group (*n*) (%)^a^			Difference in change (Δ) between groups^b^		
	Δ Baseline—8 weeks	Δ 8 weeks—12 months	Δ Baseline—12 months	Δ Baseline—8 weeks	Δ 8 weeks—12 months	Δ Baseline—12 months	Baseline—8 weeks	8 weeks—12 months	Baseline—12 months
							OR (95% CI) *p* value	OR (95% CI) *p* value	OR (95% CI) *p* value
DIGEST safety									
Improvement	4 (18%)	6 (40%)	7 (41%)	14 (56%)	4 (18%)	10 (46%)	0.24 (0.06–0.89) *p* = 0.033	1.36 (0.39–4.67) *p* = 0.63	1.10 (0.33–3.75) *p* = 0.87
No change	12 (55%)	6 (40%)	8 (47%)	9 (36%)	13 (59%)	8 (36%)			
Worsening	6 (27%)	3 (20%)	2 (12%)	2 (8%)	5 (23%)	4 (18%)			
DIGEST efficiency									
Improvement	6 (27%)	7 (47%)	7 (41%)	10 (40%)	8 (36%)	9 (41%)	0.69 (0.23–2.08) *p* = 0.51	1.59 (0.46–5.55) *p* = 0.46	1.16 (0.39–3.48) *p* = 0.78
No change	11 (50%)	7 (47%)	7 (41%)	10 (40%)	8 (36%)	11 (50%)			
Worsening	5 (23%)	1 (7%)	3 (18%)	5 (20%)	6 (27%)	2 (9%)			
DIGEST score									
Improvement	8 (36%)	6 (40%)	7 (41%)	13 (52%)	8 (36%)	11 (50%)	0.69 (0.21–2.32) *p* = 0.55	1.48 (0.48–4.61) *p* = 0.49	1.66 (0.64–4.31) *p* = 0.30
No change	10 (46%)	7 (47%)	9 (53%)	9 (36%)	7 (32%)	10 (46%)			
Worsening	4 (18%)	2 (13%)	1 (6%)	3 (12%)	7 (32%)	1 (5%)			

Abbreviations: CI = confidence interval; OR = odds ratio.

^a^ Percentage values are rounded; therefore, do not always sum to 100.

^b^ Analyses were performed using ordinal logistic regression adjusted for baseline values. Missing data were handled by multiple imputation including baseline variables related to the outcome and its missingness.

### 3.4 Subgroup Analyses

The analyses of the two subgroups (PAS score ≥ 5 or overall DIGEST score ≥ 2 at baseline FEES) revealed no statistically significant differences in the change in PAS scores from baseline to 12 months, baseline to 8 weeks, or 8 weeks to 12 months (Table 5). In accordance with the main analysis, there were statistically significant differences in the DIGEST safety score between baseline and 8 weeks for both subgroups, reflecting an improvement in the control group (*p* = 0.02 and *p* = 0.0035, respectively; Table 6). Subgroup analyses were also performed for the EAT‐10 scores, which showed no statistically significant differences for any study period.

**TABLE 5:** Comparison of change in Penetration Aspiration Scale (PAS) scores and Eating Assessment Tool (EAT)‐10 scores for the two subgroups.

Subgroup	Variable	Intervention group			Control group			Difference in change (Δ) between groups^a^		
		Δ Baseline—8 weeks	Δ 8 weeks—12 months	Δ Baseline—12 months	Δ Baseline—8 weeks	Δ 8 weeks—12 months	Δ Baseline—12 months	Baseline—8 weeks	8 weeks—12 months	Baseline—12 months
		Mean ± SD median (min‐max) *n*	Mean ± SD median (min‐max) *n*	Mean ± SD median (min‐max) *n*	Mean ± SD median (min‐max) *n*	Mean ± SD median (min‐max) *n*	Mean ± SD median (min‐max) *n*	*p*	*p*	*p*
PAS score ≥ 5 at baseline FEES	PAS	−1.7 ± 1.9 −2.0 (−4.0–1.0) *n* = 9	−0.6 ± 2.2 0.0 (−4.0–2.0) *n* = 8	−3.1 ± 2.3 −3.0 (−7.0–0.0) *n* = 9	−2.1 ± 2.0 −2.0 (−6.0–1.0) *n* = 16	0.6 ± 2.9 0.5 (−4.0–6.0) *n* = 14	−1.6 ± 2.2 −2.0 (−5.0–2.0) *n* = 14	0.67	0.36	0.17
DIGEST score ≥ 2 at baseline FEES	PAS	0.3 ± 2.3 0.5 (−4.0–4.0) *n* = 16	−0.6 ± 1.9 0.0 (−4.0–2.0) *n* = 11	−0.7 ± 2.5 0.0 (−5.0–3.0) *n* = 11	−1.4 ± 2.7 −2.0 (−6.0–4.0) *n* = 19	0.9 ± 2.5 0.5 (−3.0–6.0) *n* = 16	−0.8 ± 2.5 −0.5 (−5.0–4.0) *n* = 16	0.052	0.13	1.00

Abbreviations: FEES = flexible endoscopic evaluation of swallowing; SD = standard deviation.

^a^ Analyses were performed on complete cases using the Mann–Whitney *U*‐test for not normally distributed continuous variables.

**TABLE 6:** Comparison of dynamic imaging grade of swallowing toxicity for flexible endoscopic evaluation of swallowing (DIGEST‐FEES) safety and efficiency scores and overall DIGEST scores for the two subgroups.

Subgroup	Variable	Intervention group *n* (%)^a^			Control group *n* (%)^a^			Difference in change (Δ) between groups^b^		
		Δ Baseline—8 weeks	Δ 8 weeks—12 months	Δ Baseline—12 months	Δ Baseline—8 weeks	Δ 8 weeks—12 months	Δ Baseline—12 months	Baseline—8 weeks	8 weeks—12 months	Baseline—12 months
								*p*	*p*	*p*
PAS score ≥ 5 at baseline FEES	DIGEST safety									
	Improvement	4 (44%)	4 (50%)	7 (78%)	14 (88%)	3 (21%)	9 (64%)	0.020	0.28	0.26
	No change	4 (44%)	2 (25%)	2 (22%)	2 (13%)	6 (43%)	2 (14%)			
	Worsening	1 (11%)	2 (25%)	0 (0%)	0 (0%)	5 (36%)	3 (21%)			
	DIGEST efficiency									
	Improvement	3 (33%)	3 (38%)	4 (44%)	7 (44%)	4 (29%)	5 (36%)	0.50	0.36	0.98
	No change	4 (44%)	4 (50%)	3 (33%)	7 (44%)	5 (36%)	7 (50%)			
	Worsening	2 (22%)	1 (13%)	2 (22%)	2 (13%)	5 (36%)	2 (14%)			
	DIGEST score									
	Improvement	5 (56%)	3 (38%)	5 (56%)	11 (69%)	4 (29%)	8 (57%)	0.51	0.38	0.83
	No change	3 (33%)	3 (38%)	4 (44%)	4 (25%)	3 (21%)	5 (36%)			
	Worsening	1 (11%)	2 (25%)	0 (0%)	1 (6%)	7 (50%)	1 (7%)			
DIGEST score ≥ 2 at baseline FEES	DIGEST safety									
	Improvement	3 (19%)	4 (36%)	4 (36%)	14 (74%)	2 (13%)	8 (50%)	0.0035	0.18	0.94
	No change	10 (63%)	5 (46%)	6 (55%)	4 (21%)	9 (56%)	4 (25%)			
	Worsening	3 (19%)	2 (18%)	1 (9%)	1 (5%)	5 (31%)	4 (25%)			
	DIGEST efficiency									
	Improvement	5 (31%)	5 (46%)	5 (46%)	10 (53%)	5 (31%)	7 (44%)	0.33	0.17	0.66
	No change	8 (50%)	5 (46%)	3 (27%)	6 (32%)	5 (31%)	7 (44%)			
	Worsening	3 (19%)	1 (9%)	3 (27%)	3 (16%)	6 (38%)	2 (13%)			
	DIGEST score									
	Improvement	7 (44%)	3 (27%)	5 (46%)	13 (68%)	4 (25%)	9 (56%)	0.31	0.36	0.84
	No change	9 (56%)	6 (55%)	6 (55%)	5 (26%)	5 (31%)	6 (38%)			
	Worsening	0 (0%)	2 (18%)	0 (0%)	1 (5%)	7 (44%)	1 (6%)			

Abbreviation: FEES = flexible endoscopic evaluation of swallowing.

^a^ Percentage values are rounded; therefore, they do not always sum to 100.

^b^ Analyses were performed on complete cases using the Mantel–Haenszel chi‐square test for ordered categorical variables.

### 3.5 Intra‐ and Inter‐Rater Reliability

The intra‐rater reliability was almost perfect for PAS scores, the primary outcome, and DIGEST scores, a secondary outcome (kw [weighted kappa] = 0.9 and kw = 0.94, respectively). The inter‐rater reliability was moderate for PAS scores (kw [weighted kappa] = 0.55) and substantial for DIGEST scores (kw [weighted kappa] = 0.69).

## 4 Discussion

The purpose of this randomized study was to evaluate the rehabilitation of radiation‐induced dysphagia with the HLE over 12 months using the PAS, EAT‐10, and DIGEST‐FEES. To the best of the authors' knowledge, this is the only randomized study of its kind among HNC survivors. The results broadly demonstrated no improvement in swallowing function between baseline and the 12‐month follow‐up, rather indicating a deterioration in swallowing safety in the intervention group during the 8‐week intervention period with the HLE, which then recovered over the subsequent 12‐month maintenance period during which participants performed the HLE less frequently. We previously reported that, over the 8‐week intervention period, EAT‐10 scores improved to a greater extent in the intervention group than in the control group [33]. However, this trend was not sustained through to the 12‐month follow‐up. These results are in line with the previously published HRQL results up to 12 months [35]. Lack of self‐perceived improvement of swallowing function could explain the low adherence to training during the maintenance period; limited follow‐up contact with study SLPs is another possible explanation. As such, the results of this analysis are in line with the findings of our previous publications from this RCT.

As previously mentioned other RCTs evaluating strength based interventions for this patient group showed only minor improvements, no effects or adverse results [20, 21, 22, 23]. And, based on the combined evidence of this RCT, the HLE does not seem to improve swallowing function in individuals with radiation‐induced dysphagia. One explanation for this is that radiation can alter affected muscles, hindering the normal mechanisms that would otherwise produce significant gains from repetitive strength‐based exercise [4]. Also, according to King, the amount of muscle stimulation provided by NMES to irradiated tissues can cause high degrees of muscle fatigue [4]. It is unclear whether the HLE induces a similar degree of muscle stimulation; however, exercise‐induced muscle fatigue is one explanation for the greater improvement in swallowing safety in the control group compared to the intervention group during the 8‐week intense exercise period.

This study did not examine changes in muscle strength. However, there was no significant difference in DIGEST‐efficiency score at any time point. Also, the 8‐week follow‐up using videofluoroscopic evaluation examined kinematic variables, including hyoid bone movement, and thyrohyoid approximation during swallowing. None of these variables showed improvements in the intervention group [34]. Further, UES opening was measured, with a values between 3.8 and 7.2 mm at baseline, that is, below normal values [46, 47], and no statistically significant improvements in UES opening was seen in the intervention or the control group. A clinical study comparing the HLE to chin tuck against resistance (CTAR), a modification that enables progressive overload, found that the two methods equally increase muscle strength after intervention. However, this did not translate into improved PAS scores, residue clearance, or kinematics [15]. Such findings could be attributed to fibrotic tissue both in and surrounding key muscles involved in swallowing, which reduces their mobility [4].

The effects of tissue fibrosis on the mobility of muscles and structures involved in swallowing, as well as damage to mechanisms necessary for muscle growth with exercise [4], pose challenges when applying a strength‐based swallowing intervention for the rehabilitation of radiation‐induced dysphagia. These concerns are compounded by the added risk of adverse effects arising from intense muscle stimulation. Thus, employing intense training programs or adding progressive overload to target muscles involved in swallowing might not be the right approach for these patients. These challenges and risks are important to consider both in further research and as clinicians caring for this group of patients.

Among the participants, the time between RT completion and study inclusion ranged from 6 months to 36 months, with a mean duration of 9 months in this study. Radiation‐induced dysphagia is not stable over time, which is important to consider when interpreting the results. A meta‐analysis found that the frequency of aspiration decreases during the first 12 months after RT [12]. Nevertheless, this does not fully explain why there was an improvement in swallowing safety during the 8‐week intervention period for the control group or why the HLE training hindered improvement in the intervention group. King et al. have stressed that characteristics such as tumor location (the primary radiation site), time from dysphagia onset, and time after RT can all alter the response to rehabilitation for dysphagia, proposing the inclusion of more well‐defined groups when researching interventions for this population [4].

There is some evidence from rehabilitative interventions focusing more on respiration. Expiratory muscle strength training (EMST) has been shown to improve aspiration in patients with severe radiation‐induced dysphagia [48]. Also, a study using skill training and biofeedback to teach patients with radiation‐induced dysphagia to swallow during the optimal phase of respiration showed improvements in PAS scores, kinematics, and self‐reported dysphagia [49]. Further studies of EMST, exploring skill‐based intervention alternatives and examining the timing and coordination aspects of radiation‐induced dysphagia, are avenues for future research.

A strength of this study is that it employed both the DIGEST‐FEES and the PAS. Given that the ordinality of PAS has been questioned by Steele et al. [40], the DIGEST‐FEES protocol offers ordinal scales for evaluating swallowing safety, efficiency, and overall function. Although the DIGEST‐FEES does not change the order of the scores in the PAS as Steele et al. have suggested, it does aim to address similar difficulties by grouping scores together and weighing in the frequency of scores received [50]. The supplementary descriptive analyses using Steele's categorization of PAS scores reflect a clearer improvement in the control group, stretching from baseline to 12 months. Further, the study is strengthened by the high retention of participants (80%) at the 12‐month follow‐up.

A limitation of this study is that the inter‐rater reliability of PAS scores, the primary outcome measure, was only moderate. The intra‐rater reliability of PAS scores, however, was almost perfect, which is the basis for the results presented in the study. On the other hand, DIGEST scores proved to be a more reliable measure with substantial inter‐rater and almost perfect intra‐rater reliability. Another limitation is that there was a statistical difference in comorbidity levels according to the ACE‐27 between the control and intervention groups at baseline. Ideally, the groups should be as similar as possible. However, the higher level of comorbidity in the control group would not be expected to skew the results in favor of the intervention group. Additionally, participants were instructed to do at least 3 but could choose to do up to seven training sessions per week during the maintenance period. This contributed to a large variation in the amount of training performed, although adherence was low. Further studies should employ more definite instructions to evaluate the effects of maintenance training. The study also includes participants with several different tumor locations as well as different treatment modalities. Further, the Shaker HLE was originally developed targeting improved UES opening. Impaired UES opening was not an inclusion criteria in this study, meaning this study evaluated a broader cohort of HNC patients with dysphagia following radiotherapy. As noted, there was also a 30‐month difference in time span from the completion of RT to study inclusion. Although these variables did not differ significantly between the intervention and control groups, they could have affected the response to treatment and are important to consider when interpreting the results.

## 5 Conclusion

The HLE does not seem to improve swallowing function in individuals with radiation‐induced dysphagia when applied broadly, without targeting specific UES impairment phenotypes. Considering the collective evidence from publications of this RCT, the HLE may not be appropriate for broad dysphagia rehabilitation in this population.

## Funding

This study was funded by the Operational Healthcare Committee, Region Västra Götaland, the Swedish Cancer Society, the Department of Otorhinolaryngology at Sahlgrenska University Hospital, the Assar Gabrielsson Foundation, and by grants from the Swedish state under the agreement between the Swedish Government and the County Councils, the ALF‐Agreement.

## Ethics Statement

The study was approved by the Regional Ethical Review Board in Gothenburg, Sweden.

## Consent

All participants gave their written informed consent before inclusion.

## Conflicts of Interest

The authors declare no conflicts of interest.

## Supporting information


**Table S1:** Change in PAS categories according to Steele et al. 2017 between baseline and 8 weeks, 8 weeks and 12 months and baseline and 12 months.

## Data Availability

Research data are not shared.
