# Supplementary material for: Treatment With the Shaker Head‐Lift Exercise in Head and Neck Cancer Patients With Radiation‐Induced Dysphagia: 12‐Month Results on Swallowing Function From a Randomized, Controlled Trial
Source: Head Neck. 2026 Jun 14;48(12):3263–76. doi: 10.1002/hed.70352 (PMC13755744; doi:10.1002/hed.70352)
Supplement: Supplementary file 1 [file HED-48-3263-s001.docx]

**Supplementary table 1** Change in PAS categories according to Steele et. al. 2017 between baseline and 8 weeks, 8 weeks and 12 months and baseline and 12 months.

| **Variable** | | **Baseline** | | | **8 weeks** | | | **12 months** | |
| --- | --- | --- | --- | --- | --- | --- | --- | --- | --- |
|  | **Intervention**  **n (29)** | | **Control**  **n (28)** | **Intervention**  **n (22)** | | **Control**  **n (26)** | **Intervention**  **n (17)** | | **Control**  **n (22)** |
| **PAS category** | **n (%)*** | | **n (%)*** | **n (%)*** | | **n (%)*** | **n (%)*** | | **n (%)*** |
| A | 8 (28%) | | 7 (25%) | 5 (23%) | | 8 (31%) | 6 (35%) | | 8 (36%) |
| B | 14 (48%) | | 13 (46%) | 14 (64%) | | 12 (46%) | 10 (59%) | | 11 (50%) |
| C | 3 (10%) | | 5 (18%) | 2 (9%) | | 2 (8%) | 1 (6%) | | 2 (9%) |
| D | 4 (14%) | | 3 (11%) | 1 (5%) | | 4 (15%) | 0 (0%) | | 1 (5%) |
|  | **Δ Baseline - 8 weeks** | | | **Δ 8 weeks – 12 months** | | | **Δ Baseline – 12 months** | | |
| No change | 13 (45%) | | 12 (43%) | 10 (45%) | | 11 (42%) | 6 (35%) | | 9 (41%) |
| Improvement | 5 (17%) | | 9 (32%) | 4 (18%) | | 6 (23%) | 5 (29%) | | 10 (45%) |
| Worsening | 5 (17%) | | 4 (14%) | 1 (5%) | | 5 (19%) | 3 (18%) | | 3 (14%) |
| ^*^ Percentages rounded, therefore does not always sum to 100. | | | | | | | | | |
